# Pediatric Scurvy: When Contemporary Eating Habits Bring Back the Past

**DOI:** 10.3389/fped.2018.00126

**Published:** 2018-05-01

**Authors:** Alice Brambilla, Cristina Pizza, Donatella Lasagni, Lucia Lachina, Massimo Resti, Sandra Trapani

**Affiliations:** ^1^Pediatric Department, University of Florence, Anna Meyer Children Hospital, Florence, Italy; ^2^Clinical Pediatric Department, Anna Meyer Children Hospital, Florence, Italy

**Keywords:** scurvy, vitamin C deficiency, malnutrition, eating disorders, pediatric nutrition

## Abstract

Vitamin C deficiency is anecdotal in developed countries, mainly associated with underling clinical morbidities as autism or neurological impairment. Chronic insufficient dietary supply is responsible for vascular fragility and impaired bone formation, resulting in gingival bleeding, petechial lesions, articular and bone pain or limb swelling. Children may present anorexia, irritability, failure to thrive, limping or refusal to walk. Accordingly, pediatric scurvy is frequently misdiagnosed with osteomyelitis, septic arthritis, bone and soft tissue tumor, leukemia, bleeding disorders, and rheumatologic conditions. We report the case of a 3-years old child developing scurvy as consequence of strict selective diet; extensive and invasive investigations were undertaken before the correct diagnosis was considered. Despite being considered a rare condition, scurvy still exists nowadays, even in children with no apparent risk factors living in wealthy families. The increasing popularity of dietary restriction for children, especially those with allergies, may potentially enhance the occurrence of scurvy in apparently healthy children. Appropriate dietary anamnesis is fundamental in order to highlight potential nutritional deficit and to avoid unnecessary invasive diagnostic procedures. Patients without considerable risk factors may benefit from psychological support in order to investigate possible eating disorders.

## Background

Scurvy is a well-defined clinical condition secondary to chronic ascorbic acid deficiency, currently anecdotal in developed countries. Neurological disorders, autism, iron overload due to multiple transfusions, chemotherapy, bone marrow transplant and hemodialysis are considered the main predisposing conditions in modern age (1, 2). The diagnosis is often misleading, since other morbidities as malignancies, coagulopathies, septic arthritis, osteomyelitis or rheumatologic disorders are often considered at first (3–5). We report the case of an otherwise healthy child who developed scurvy as consequence of strictly selective diet; the patient underwent extensive and invasive examinations before the correct diagnosis was considered.

## Case presentation

A 3-years Russian boy was admitted to our hospital for intense pain and recurrent swelling at left thigh, resulting in persistent refusal to walk. Medical history revealed a strict selective diet since weaning, mainly based on grain, rice and corn with almost complete avoidance of meat, fish, fruit and vegetables. Inadequate eating habits derived from child's refusal to try new food. Dairy products were also avoided due to presumed milk allergy; no vitamin supplementation was administered.

Pathologic anamnesis documented the recurrence of nummular, erythematous and itchy rash from the age of 18 months onwards. At 2 years of age, also lower limbs arthralgia appeared, followed by left thigh swelling, refusal to walk and to eat. Due to progressive worsening in clinical conditions with severe pallor, dehydration, fever and serious asthenia, the child was hospitalized in his native country, where septic shock was initially suspected. He received blood cell transfusions, albumin and immunoglobulin infusion, antibiotics, inotropes and respiratory assistance. Dietary intake was provided through nasogastric tube and parenteral support; unfortunately, no data regarding nutritional supply are available. Lower limb CT-scan was performed, documenting areas of bone rarefaction and reabsorption, along with periosteal reaction and soft tissues edema. Bone biopsy excluded the hypothesis of osteomyelitis, suggesting instead the diagnosis of myosarcoma. Further hospitalization at Oncological Department followed, where complete laboratory/radiological work-up, bone marrow aspiration and additional muscular and bone biopsy definitively excluded malignancies. Magnetic Resonance imaging (MRI) highlighted focal changes in bone marrow of pelvic bones and thighs, soft tissue swelling and periosteal infiltration of femurs. A second muscular biopsy was undertaken, concluding for not specific “proliferative myositis and productive vasculitis.” Initial parenteral nutrition was administered, followed by enteral nutrition trough nasogastric tube. He was discharged in good general conditions and almost complete clinical remission. Afterwards, the child returned to habitual selective diet. After two months, arthralgia, intense pain and swelling of left tight with refusal to walk reappeared, requiring a second hospitalization course. Coagulation profile and Doppler ultrasound scan excluded thrombotic processes; severe osteoporosis was documented at bone densitometry. Due to the persistence of symptoms, he came to our hospital for second-opinion.

## Laboratory investigations and diagnostic tests

**Figure 1:**
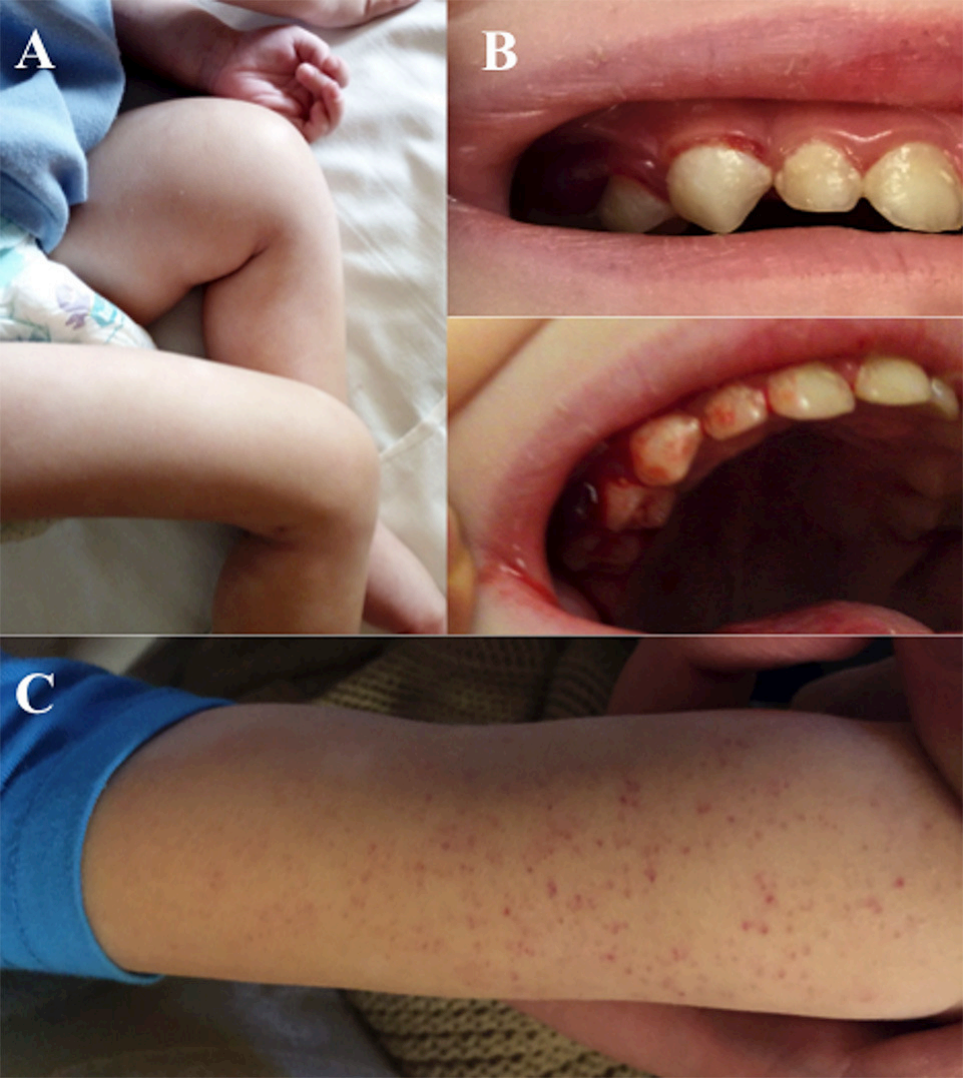
Patient's clinical features on admission: swelling and antalgic posture of left tight **(A)**, pigmented and easy bleeding gums **(B)**, petechial lesions **(C)**.

**Figure 2:**
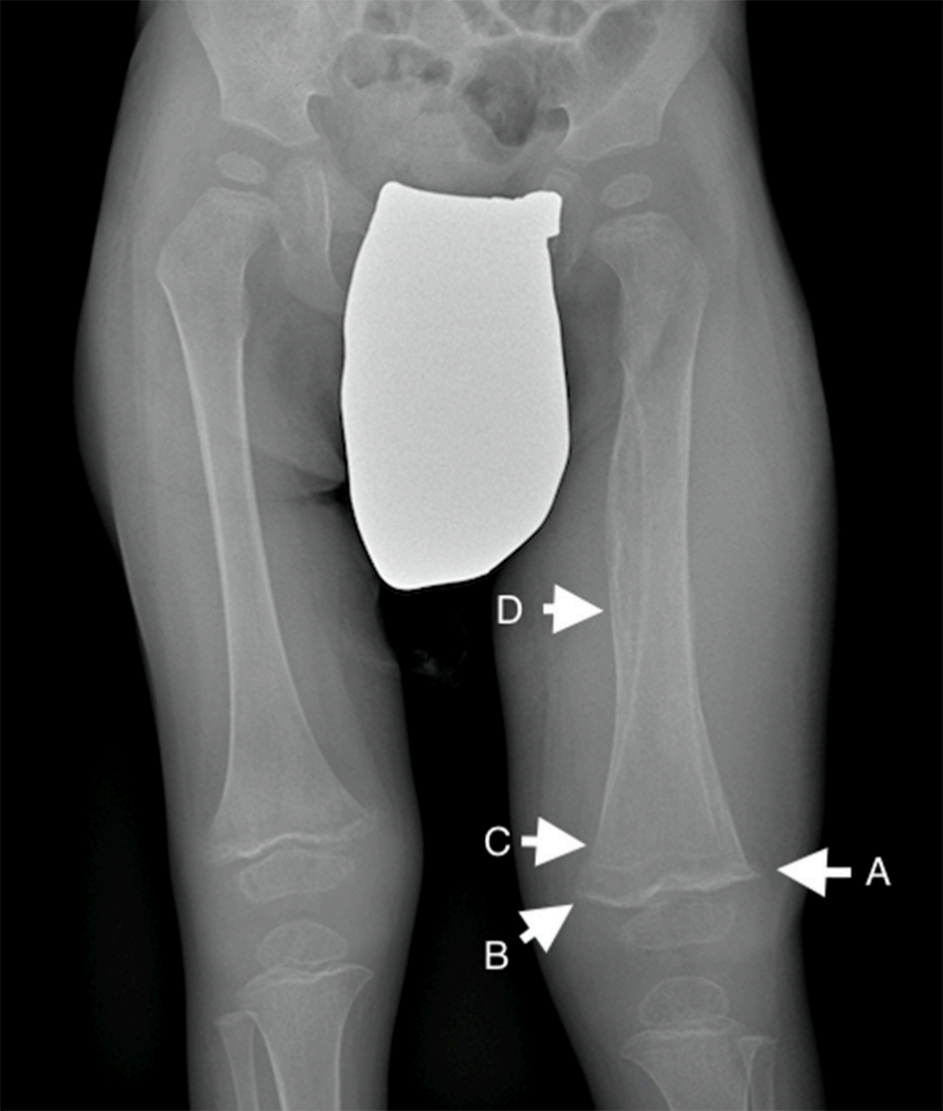
Lower limb X-Ray showing generalized osteopenia, metaphyseal spurs with concomitant cupping of the metaphysis (“Pelken spurs”, arrow A), lucent metaphyseal bands (“Trümmerfeld zone”; arrow B) and dense metaphyseal line (“Frankel's line”; arrow C). Extended periosteal reaction could also be documented (arrow D).

At admission, child's weight was 12 kg (<5° percentile), height 92 cm (5–10° percentile) and BMI 14,2 kg/m^2^ (<5°). Dietary anamnesis highlighted selective and unbalance diet, mainly based on carbohydrate (rice, corn, grain, potatoes). Considering what reported by parents, we esteemed daily caloric intake ranging from 450 to 500 kcal/day [minimum for age: 880 kcal/day 1)]. Habitual diet resulted low in fat [10–15% of total calories; recommended intake: 35–40% of total calories 1)] and proteins [0.5–0.7 g/kg/day, recommended intake: 1 g/kg/day 1)]. Protein intake mainly derived from legumes (beans, lentils, peas), whereas meat and fish were almost completely avoided, as well as dairy products. Fresh fruits and vegetables were rarely administered, since the child didn't like them and parents decided not to force him. Clinical examination revealed poor nutritional status, with severe pallor and irritability. Swollen and bleeding gums, diffuse petechiae, swollen and intense pain at left tight could be documented (Figure 1. Both parents have signed written consent form for sensible data and patient's picture; standard form approved by ethical committee our institution has been utilized at this purpose). Laboratory exams revealed low levels of Vitamin A and D, along with severe ascorbic acid deficiency (0.17 micromol/L, normal value >26.14). Multiple food allergies (milk, egg, peanut, cod, salmon, sole) were confirmed at skin prick test. Femoral X-ray documented extended periosteal reaction, metaphyseal spurs with concomitant cupping of the metaphysis (Pelken spurs), lucent metaphyseal bands (Trümmerfeld zone) and dense metaphyseal line (Frankel's line) more evident in left femur (Figure 2). During the first days of hospitalization the child required blood cell transfusion due to severe anemia, nasogastric tube to support nutrition and analgesics. Enteral feeding was initially based on hypoallergenic amino-acids formula (1000 kcal/L; protein 33 g/L; carbohydrates 104 g/L; fat 50 g/L, MCT 35%; osmolality 590 mOsm/kg). As to avoid refeeding syndrome, we decided to start from minimum caloric intake (475 kcal/day, equal to 40 kcal/kg/day), subsequently increased up to 100 ml/kg/day. Continuous feeding was switched to cyclic infusion (200 ml for 6 meals/day) within few days, given good child's tolerance. Appropriate micronutrients intake was ensured, as recommended for age and weight (1) (Vitamin A 300 mcg/day, Vitamin D 15 mcg/day, Vitamin B12 0.9 mcg/day, Folate 140 mcg/day, Calcium 700 mg/day, Iron 8 mg/day). Vitamin C supplementation was added, starting from 500 mg/day for six days, followed by 300 mg/day in maintaining phase. Progressive improvement in general conditions was documented, with resolution of cutaneous and mucosal bleeding, gain of weight (600 g in 2 weeks), reduction of pain and swelling and amelioration in walking impairment. After 12 days of treatment, vitamin C level markedly increased to 79.09 micromol/L. The evidence of reduced serum vitamin C levels along with the prompt response to vitamin C supplementation confirmed the clinical suspicion of pediatric scurvy; characteristic radiological findings corroborated the final diagnosis. Psychological evaluation highlighted a food behavioral disorder as possible cause of patient's selective diet, probably secondary to previous traumatic detachment from maternal figure. Transition from nasogastric-tube to oral feeding was difficult and lasted several days; in this clinical setting, psychological support was mandatory in order to facilitate child's acceptance of oral food intake.

## Discussion

In the modern age, scurvy has been reported in poor countries (2, 3) or in children with underlying chronic conditions as developmental delay, autism, iron overload secondary to repeated transfusions, chemotherapy, bone marrow transplant and hemodialysis (4, 5). Beside this, the vogue to pursue selective and/or unbalanced diets is spreading, with a consistent risk of micronutrient malnutrition even in apparent healthy children.

Humans, unable to produce Vitamin C, are entirely dependent on dietary supply. Its chronic deficiency leads to impaired collagen synthesis, determining vascular fragility and abnormal formation of osteoid tissue and dentin, but also affects hormonal synthesis and immune regulation.

After 1–3 months of ascorbic acid deficiency, fatigue, irritability, malaise and anorexia appear. Skin hyperkeratosis, corkscrew hair, petechial hemorrhages, purpura and swollen bleeding gums are common findings. In childhood, musculoskeletal symptoms as bone pain, arthralgia, limping, refusal to walk, limb and joint swelling or pseudo-paralysis are typical presenting signs (6–8).

Affected patients usually undergo extensive laboratory and radiological workup, and various misdiagnoses, including osteomyelitis, septic arthritis, malignancies, autoimmune diseases, venous thrombosis, bleeding disorders and child abuse, are commonly made (9–12). Interestingly, most of these conditions have previously been suspected in our case, too.

Serum determination of vitamin C levels is the gold standard for the diagnosis. Radiological examinations are complementary and reveal osteopenia, metaphyseal rarefaction, metaphyseal spurs secondary to healing fractures, metaphyseal dense line of cartilage calcification and round sclerotic cortex surrounding growth centers in the epiphysis (4, 9). As in our case, periosteal elevation and edema associated with swelling in adjacent soft tissue can also be documented. MRI scan may highlight non-specific focal changes in bone marrow, associated with bilateral increased lower-extremity metaphyseal signal changes and periosteal reaction (13).

No specific regimen of treatment is defined in pediatric scurvy. Oral administration is adequate even in the most severe cases. Dosages can range from 100–300 mg to 1000 mg/day in childhood. In our case, a dose of 500 mg/day for six days, followed by 300 mg/day in maintaining phase was administered, with dramatic clinical improvement within few days. Quick clinical response may be considered as ex-juvantibus diagnostic criteria (9, 14).

Psychological evaluation was useful to understand the reason of child's feeding behavior, highlighting an abnormal relationship between mother and son and a related traumatic detachment event. Documented food allergic reactions probably worsened this condition.

## Concluding remarks

Scurvy still exists nowadays, even in children with apparent no risk factors living in wealthy families. The increasing popularity of dietary restriction for children, especially those with allergies, may potentially enhance the occurrence of scurvy in presumably healthy children.

Appropriate dietary anamnesis is fundamental in order to highlight potential nutritional deficit and to avoid unnecessary invasive diagnostic procedures. We suggest collecting a detailed dietary history in all children, with special attention to caloric, macro- and micronutrients intakes, since adequate diet influences child's growth, cognitive development and diseases prevention. Psychological support may be helpful to exclude abnormal eating disorders in selected clinical setting.

## Author contributions

AB, CP, and ST gave a substantial contribution in article conception and design. LL, DL, and MR participated in acquisition of data. AB, CP, and DL drafted the manuscript; ST, LL, and MR critically revised it. All the authors gave their final approval to this manuscript and agree to be accountable for all aspects of work ensuring integrity and accuracy.

### Conflict of interest statement

The authors declare that the research was conducted in the absence of any commercial or financial relationships that could be construed as a potential conflict of interest.
